# A New *Oidiodendron maius* Strain Isolated from *Rhododendron fortunei* and its Effects on Nitrogen Uptake and Plant Growth

**DOI:** 10.3389/fmicb.2016.01327

**Published:** 2016-08-23

**Authors:** Xiangying Wei, Jianjun Chen, Chunying Zhang, Dongming Pan

**Affiliations:** ^1^College of Horticulture, Fujian Agriculture and Forestry UniversityFuzhou, China; ^2^Department of Environmental Horticulrture and Mid-Florida Research and Education Center, Institute of Food and Agricultural Sciences, University of Florida, ApopkaFL, USA; ^3^Shanghai Academy of Landscape Architecture Science and PlanningShanghai, China

**Keywords:** ammonium transporters, ericoid mycorrhiza, nitrate transporters, nitrogen, *Oidiodendron maius*, *Rhododendron*

## Abstract

A new mycorrhizal fungal strain was isolated from hair roots of *Rhododendron fortunei* Lindl. grown in Huading Forest Park, Zhejiang Province, China. Morphological characterization and internal transcribed spacer rDNA analysis suggested that it belongs to *Oidiodendron maius* Barron, and we designated it as strain Om19. Methods for culturing Om19 were established, and the ability of Om19 to form mycorrhizae on *R. fortunei* was evaluated in a peat-based substrate. Microscopic observations showed hyaline hyphae on the surface of hair roots and crowded hyphal complexes (hyphal coils) inside root cortical cells of *R. fortunei* after inoculation, indicating that the roots were well colonized by Om19. In a second experiment, fresh and dry weight of *R. fortunei* 2 months after Om19 inoculation were greater than uninoculated plants, and the total nitrogen absorbed by plants inoculated with Om19 was greater than the uninoculated controls. qRT-PCR analysis of five genes related to N uptake and metabolism (two nitrate transporters, an ammonium transporter, glutamine synthetase, and glutamate synthase) showed that these genes were highly upregulated with twofold to ninefold greater expression in plants inoculated with Om19 compared to uninoculated plants. In the third experiment, Om19 was inoculated into the peat-based substrate for growing Formosa azalea (*Rhododendron indica* ‘Formosa’). ‘Formosa’ azalea plants grown in the inoculated substrate had larger canopies and root systems compared to uninoculated plants. Our results show that Om19 could be an important microbial tool for improving production of *Rhododendron* plants.

## Introduction

About 6,000 recognized species of soil fungi form mutualistic symbioses with roots of more than 90% of land plants, which are collectively known as mycorrhizas (Smith and Read, 2008). The symbiosis enables plants to adapt to different soil conditions, improves roots in nutrient acquisition, enhances plant resistance to root pathogen infection, and increases plant growth (Bergero et al., 2000; Smith and Read, 2008). Mycorrhizal fungi are classified as ectomycorrhiza and endomycorrhiza depending on whether fungal hyphae colonize root intercellular spaces or the interior of root cells (Bonfante and Genre, 2010). Endomycorrhiza includes arbuscular, ericoid, and orchid mycorrhiza (Smith and Read, 2008).

Ericoid mycorrhizal (ERM) fungi specifically form symbiotic associations with roots of plants in the family Ericaceae (Perotto et al., 2012). Such ERM fungi include *Hymenoscyphus ericae* (Read) Korf and Kernan, *Oidiodendron* spp., *Cadophora finlandia* (Wang and Wilcox) Harrington and McNew, and *Scytalidium vaccinii* Dalpe, Litten and Sigler (Read, 1996). Among these, *Oidiodendron maius* Barron is one of the most widely investigated ERM fungi. Its genome has recently been sequenced (Kohler et al., 2015). The symbiosis of *O. maius* with roots of ericaceous plants is known to facilitate the exchange of nutrients (Rice and Currah, 2006). *O. maius* also play a crucial role in the protection of host plants against heavy metal toxicity (Daghino et al., 2016). *O. maius* was first identified by Barron (1962) from collections of peat soil in Canada, and then isolated from roots of an ericaceous plant in Japan (Tokumasu, 1973). Subsequently, *O. maius* has been recorded as ERM endophytes of several taxa in the Ericaceae (Hambleton and Currah, 1997; Addy et al., 2005) and is especially common in the roots of *Rhododendron* species (Usuki et al., 2003; Bougoure and Cairney, 2005; Zhang et al., 2009; Tian et al., 2011).

A characteristic of ERM fungi is their ability to improve nitrogen (N) uptake in plants (Read, 1996; Bucking and Kafle, 2015). However, there is still controversy regarding how N, and particularly which form of N, is absorbed by ericaceous plants. Plants can absorb either nitrate (NO_3_^-^) or ammonium. Cranberry (*Vaccinium macrocarpon* Ait.), a member of the family Ericaceae, was reported to be unable to take up NO_3_^-^ as a sole source of N in hydroponic culture (Rosen et al., 1990; Smith, 1993). An explanation is that cranberry has adapted to acidic soil conditions where pH ranges from 4 to 5 and nitrification is typically negligible at soil pH below 5.5 (Paul and Clark, 1989), therefore the adaptation has resulted in the loss of plant capacity to absorb NO_3_^-^. However, recent reports showed that inoculation with the fungus *Rhizoscyphus ericae* increased the capacity of cranberry to absorb NO_3_^-^ (Kosola et al., 2007). Compared to uninoculated plants, inoculating certain blueberry (*Vaccinium corymbosum* L.) cultivars with ERM fungi can increase nutrient concentration, particularly N, and plant growth (Scagel, 2005). Yin et al. (2010) also found that ERM fungi significantly increased the ability of *Rhododendron fortunei* to absorb N, especially in the form of NO_3_^-^.

Nitrate uptake is carried out by two nitrate transporter (NRT) systems: a low-affinity transport system (LATS, active at NO_3_^-^ concentrations higher than 0.2 mM) and a high-affinity transport system (HATS, operating at NO_3_^-^ concentrations lower than 0.2 mM; Malagoli et al., 2004). Ammonium is absorbed through ammonium transporters (AMTs; von Wittgenstein et al., 2014). Increased ammonium uptake triggers plant glutamine synthetase (GS) and glutamate synthase (GOGAT) activities as glutamine and glutamate play crucial roles in N metabolism. In the symbiosis of arbuscular mycorrhizal (AM) fungi with host plants, N is absorbed by the extraradical mycelia and converted into the amino acid arginine for transport into the intraradical mycelia (Gomez et al., 2009; Tian et al., 2010). After internal migration is complete, the arginine is broken down through the urease cycle into ammonium for transport into the plant (Govindarajulu et al., 2005). Thus far, whether or not ERM fungi act like AM fungi in facilitating N uptake in ericaceous plants is largely unknown.

An important effort in our mycorrhizal research program has been pursuing a better understanding of the potential of ERM fungi as biofertilizers for improving plant growth of ericaceous species. We believe that valuable *O. maius* strains could be isolated from hair roots of understorey *R. fortunei* in Chinese forest parks, and that some isolated strains could improve *Rhododendron* N uptake and growth in commercial nursery production. This report is intended to document a new strain of ERM fungi isolated from hair roots of *R. fortunei* and its identification and characterization. The effects of this new strain on plant growth, N absorption, and N metabolism related gene expression were also determined. A better understanding of ERM fungal mediated N uptake in host plants may help improve production of some economically important ericaceous crops such as blueberry, cranberry, and rhododendron.

## Materials and Methods

### Isolation of Mycorrhizal Fungi

Plants of *R. fortunei* with a height less than 40 cm that were grown singly without any plants in a radius of 2 m were identified in Huading Forest Park (29° 15′ N 121° 06′ E), Zhejiang Province, China where mixed pines (*Pinus taiwanensis*) were grown. Root samples of five identified plants were excavated from the upper 15 cm of the soil using a shovel. Most of the soil was removed from root samples by shaking, and roots were placed in plastic bags and stored at 4°C until used for fungal isolation.

Fungi were isolated from hair roots by direct plating (Stoyke and Currah, 1991; Zhang et al., 2009). Briefly, collected root samples were soaked in cool tap water and washed gently to remove soil. Hair roots removed from the root samples were surface sterilized by immersion in a 72% ethanol for 30 s, followed by immersion in 10% sodium hypochlorite for 15 min, and then rinsed four times in sterile distilled water. The sterilized hair roots were cut into 0.3–0.5 cm segments, plated on modified Melin-Norkans agar medium (MMN; Xiao and Berch, 1992), and incubated in the dark at 25°C for 3–5 weeks. A total of 100 root pieces, 20 per plant, were cultured and those producing rapidly sporulating fungi were removed. Slower growing fungi were subcultured on 2% malt extract agar (MEA; Thom and Church, 1926), a total of 84 were isolated and maintained in the dark at 25°C for 2–4 weeks for morphological identification (described below), and subcultures were also stored at 4°C until further use. Ten of the slower growing isolates were randomly selected for a preliminary evaluation of their ability to form mycorrhizae. Results from this preliminary evaluation indicated that one isolate showed promise in forming mycorrhizal association with *R. fortunei* and improving seedling growth. This isolate was selected for further molecular identification and for plant experiments described below. This isolate was later designated as Om19.

### Morphological Identification of Om19

Morphological characteristics including colony diameter, color, thickness, texture, and pigments, and reverse side of colony color were examined after Om19 was cultured on MEA for 14 days in the dark at 25°C. To induce conidia and conidiophores, a small volume (about 50 μl) of 20% potato dextrose broth was dropped to each of two holes of a glass slide placed in a 9 cm plastic Petri dish, hyphae of isolate were added, and the slides were incubated in the dark at 25°C for 7–21 days. Conidia and conidiophores were observed under a light microscope, and fungi were putatively identified with taxonomic keys in Barron (1962) and Domsch et al. (1980).

### Molecular Identification of Om19

The isolate of Om19 was grown in potato dextrose broth on a rotating shaker (150 rpm) at 25°C in the dark for 5 days. Mycelia were collected by filtrating through Whatman filter paper No. 1 and genomic DNA was isolated from the mycelia using the modified cetyltrimethylammonium bromide method (Gardes and Bruns, 1993). The internal transcribed spacer (ITS) region was amplified using the ITS1 (5′ TCCGTAGGTGAACCTG CGG 3′) and ITS4 (5′ TCCTCCGCTTATTGATATGC 3′) primers. The amplifications were performed in a 50 μl reaction volume containing 50 ng of genomic DNA, 50 pmol of each primer, 100 μM of each dATP, dGTP, dCTP, and dTTP, 1 U of Taq polymerase, and 5 μl of PCR buffer. The tubes were incubated at 95°C for 2 min and then subjected to 35 cycles as follows: 94°C for 40 s, 60°C for 40 s, and 72°C for 45 s; a final incubation was carried out for another 5 min at 72°C. The PCR products were digested with the restriction endonucleases, *Hinf*I, *Rsa*I, *Msp*I, *Bsu*RI, and *Taq*I according to the manufacturer’s instructions. The restriction fragments were separated by electrophoresis in 2.5% (w/v) agarose gels. The base pair lengths of individual fragments were determined by comparison with a 50-bp ladder. Fragments smaller than 50 bp were not scored.

The PCR products were cloned with the PMD18-T easy vector system and analyzed by an ABI 3730XI automatic DNA sequencer. The sequence was submitted to the NCBI database under the accession number KU382495. The sequences of mycorrhizal species that most closely matched the sequence of Om19 were obtained by Basic Local Alignment Search Tool (BLAST) from the GenBank database. The alignments were performed with ClustalX and then adjusted to optimize the aligned sites. The sequences and selected fungi from GenBank database were analyzed by neighbor joining using distances from Kimura’s two-parameter model with the MEGA5.0 software system. To assess support for nodes, 1,000 bootstrap replications were performed.

### Mycorrhizae Synthesis of Om19

The Om19 was examined for its ability to colonize hair roots of *R. fortunei* seedlings. Seeds of *R. fortunei* were rinsed in running tap water for 2 h and surface sterilized four times, 5 min each, using 25% (v v^-1^) solution of commercial bleach (8.25% NaOCl) followed by rinsing in sterile distilled water three times. The sterilized seeds were germinated on a half-strength Economou and Read medium (Economou and Read, 1984). The medium was supplemented with 1.5% (w v^-1^) sucrose and 0.7% (w v^-1^) agar with pH adjusted to 5.2, autoclaved at 121°C for 30 min, and 30 ml was transferred into each culture vessel (150 ml). Seeds were germinated in a culture room under a 16 h photoperiod provided by cool-white fluorescent lamps at a photon flux density of 50 μmol m^-2^ s^-1^ and a temperature of 25°C.

A peat based substrate was formulated by mixing dry Klasmann peat (Geeste, Germany) with dry sand (initially washed two times with tap water, then five times with deionized water and dried) at 2:1 ratio based on volume. The organic matter and total N content of the Klasmann peat were 965.3 g/kg and 0.89%, respectively. A MMN nutrient solution devoid of malt extract and glucose was prepared where the N source was replaced by Ca(NO_3_)_2_ with a final N concentration at 3.79 m*M* and its pH was adjusted to 5.2. The substrate was moistened with the modified MMN solution at 3:2 ratio by volume, and its pH was tested to be 5.2 by press extraction method (Scoggins et al., 2002). Twenty cylindrical vessels (400 mL) were filled with 100 mL of substrate, covered with caps, and autoclaved at 121°C for 30 min.

Two months after seed germination, seedlings were transferred under aseptic conditions to the culture vessels containing the sterilized peat-based substrate with five seedlings per vessel. Mycelium of Om19, cultured on MEA for 2 weeks, was collected using a sterile 5-mm cork borer. After extra medium beneath the top 1 mm layer of MEA with hyphae was removed, the 5-mm diameter disks were cut into half and inoculated into the peat-based substrate next to each of the five *R. fortunei* seedlings. Ten culture vessels were inoculated with Om19, and the other 10 were used as control without inoculation. The experiment was arranged as a randomized complete block design with 10 blocks (replications). Plants were grown in a culture room under the conditions described above. After 2 months, shoots were harvested from each vessel and roots were removed from substrate by rinsing with sterile deionized water. At harvest, the number of leaves and roots, and the length and fresh weight of shoots (both stem and leaves) and roots (entire roots) of each seedling were recorded, and mean of five seedlings per vessel were calculated.

Roots from one randomly selected seedling per vessel were immediately fixed in formaldehyde–acetic acid–ethanol (FAA) for 24 h and cleared at 90°C for 1 h in 10% KOH. The roots were then acidified with 1% HCl and stained in a lactophenol-trypan blue (0.05% trypan blue in lactophenol) for 5 min at 90°C (Phillips and Hayman, 1970). Stained roots were cleared with fresh lactophenol, cut to 5 mm segments, and examined under a light microscope for ERM structures. Root colonization was quantitatively assessed using the method described by Biermann and Linderman (1981) as the percentage of root length with internal hyphal coils. A minimum eight segments from the control plant and 25 segments from the Om19-inoculated plant randomly selected per vessel were examined under the light microscope. Additionally, roots of randomly selected seedlings from the two treatments were observed under scanning electronic microscope (SEM) using the method described by Bonfante-Fasolo and Gianinazzi-Pearson (1979). All specimens were coated with gold and platinum and examined using a SEM at 20 kV.

### Effects of Om19 on Plant Growth and N Uptake

Two experiments were performed to determine the effect of Om19 on *Rhododendron* growth and N uptake. In the first experiment, 60 culture vessels (400 ml) were filled with 100 ml of a peat-based substrate prepared as described above. Two-month old seedlings of *R. fortunei* were transplanted into culture vessels (five per vessel), and 30 vessels were inoculated with Om19 as described above. The remaining 30 were uninoculated as the control treatment. The experiment was arranged as a randomized complete block design with five blocks, and each treatment had six vessels per block. Plants were grown in a culture room under the conditions described above without supplying any additional nutrients. Two months after inoculation, seedlings were collected by gently washing away of substrate from roots. A root with a length of 3 cm was sampled from seedlings of five randomly selected vessels per treatment and used for examining Om19 colonization as described above. Fresh weight of seedlings from each block (30 seedlings) was recorded after blotting with paper towel, and dry weights were also determined after oven-drying at 80°C for 48 h. Substrate pH at the end of the experiment was also recorded using the press extraction method. The seedlings (30 seedlings together) from each block were analyzed for N content using CNS Auto-Analyzer (VarioMAX, Elementar Americas, Mt. Laurel, NJ, USA), and total N per 30 seedlings was calculated.

In a second experiment, Om19 was evaluated in a greenhouse with commercial production practices. Rooted cuttings of a popular Formosa azalea (*Rhododendron indica* ‘Formosa’) were potted in a peat-based substrate (peat and sand at 2:1 ratio by volume) in 20 10-cm diameter containers (0.5 L). The Om19 inoculum was prepared by transferring 6 plugs (5-mm-diameter) of MEA culture of Om19 into 1 L of liquid MEA and incubating in a rotary shaker (1,500 rpm) at 25°C for 10 days in the dark. The culture was fragmented with a blender, diluted with sterile deionized water in a 1:1 ratio, and inoculated by pouring the inoculum mix onto the substrate of 10 containers, 10 ml each. Plants in the other 10 containers were used as controls by receiving 10 ml of 50% of diluted MEA without the inoculum. The experiment was set as a randomized complete block design with 10 blocks. A week after transplanting, all plants were fertigated with Peters Professional 20–20–20 General Purpose Fertilizer (Scotts Co., Marysville, OH, USA) with N at 100 mg L^-1^ and every other week thereafter. Plants were grown in a shaded and evaporative pad cooled greenhouse under a maximum photosynthetically active radiation of 285 μmol m^-2^ s^-1^. Temperatures ranged from 18.3 to 32.2°C and relative humidity from 50 to 80%. After 4 months, the number of leaves and canopy height of each plant were recorded, and substrate was removed from roots by washing with tap water. Plants were photographed while roots were immersed in water.

### qRT-PCR Analysis of Genes Related to N Metabolism

Expression of some key genes related to N uptake and N metabolism in Om19 inoculated and uninoculated plants was investigated in an experiment similar to the above culture room experiment with *R. fortunei*. Seedlings of *R. fortunei* were grown in 400 ml vessels containing the sterilized peat-based substrate described above. A total of 120 vessels were prepared in order to collect enough root tissue for RNA extraction. Sixty vessels were inoculated with Om19 and the rest were uninoculated as controls. The experiment was arranged as a randomized complete block design with three blocks and each treatment was replicated 20 times per block. Plants were grown in a culture room as described above. A root with a length of 3 cm was sampled from seedlings of five randomly selected vessels per treatment and assessed for root colonization as describe above. All roots were harvested 2 months after inoculation, and total RNAs were extracted with TRIzol reagent (Invitrogen, USA) and treated with RNase-free DNase I (Takara Biotechnology, China). qRT-PCR was carried out to analyze the expression level of *RfNRT1-1* and *RfNRT1-2* (NRTs), *RfAMT* (ammonium transporter), *RfGS* (GS), and *RfGOGAT* (glutamate synthase). These genes were isolated from roots of *R. fortunei*, and their sequences were submitted to the GenBank with accession numbers KX506094, KX506095, KX506096, KX506097, and KX506098 for *RfAMT, RfNRT1-1, RfNRT1-2, RfGS*, and *RfGOGAT*, respectively. The sequence homology of these genes to those in other plant species is presented in Supplementary Table S1. Primers specifically for *R. fortunei* were designed according to the cDNAs with Primer Premier software (version 5.0; Supplementary Table S2). EF1α was used as an internal control gene. The first strand cDNA was synthesized using the PrimeScript first cDNA Synthesis Kit (Takara, Dalian, China). qRT-PCR was performed in a 20 μL reaction mixture containing 2x SYBR Master Premix Ex Taq II 12.5 μL (Takara, Dalian, China), 1 μL of cDNA template (1:5 dilution), and 1 μL of each corresponding primer for the genes of interest and EF1α. qRT-PCR of three biological replicates (20 seedlings from each block as a replicate) for each sample was performed for 5 s at 95°C, 10 s at 56°C, and 20 s at 72°C using a LightCycler 480 II System (Roche Applied Science, Indianapolis, IN, USA). The relative expression levels were normalized and calibrated according to the 2^-ΔΔCT^ method (Schmittgen and Livak, 2008) where EF1α was an internal reference and seedlings before Om19 inoculation was control. For a given gene, the relative expression level was expressed as mean ± SE with three replicates.

### Data Analysis

Plant growth data from mycorrhizae synthesis and fresh and dry weight as well as N content data from plant-growth experiments were analyzed separately by experiments using a mixed model where treatment effects were considered fixed and block effects considered random. After checking for normality and homogeneity of variances using SPSS Statistics (SPSS Inc., Chicago, IL, USA), all data were subjected to analysis of variance using SPSS 19.00 statistical software, and the significance probability for treatment effects was evaluated at *P* ≤ 0.05 or 0.01 level.

## Results

### Morphological Characteristics

**FIGURE 1:**
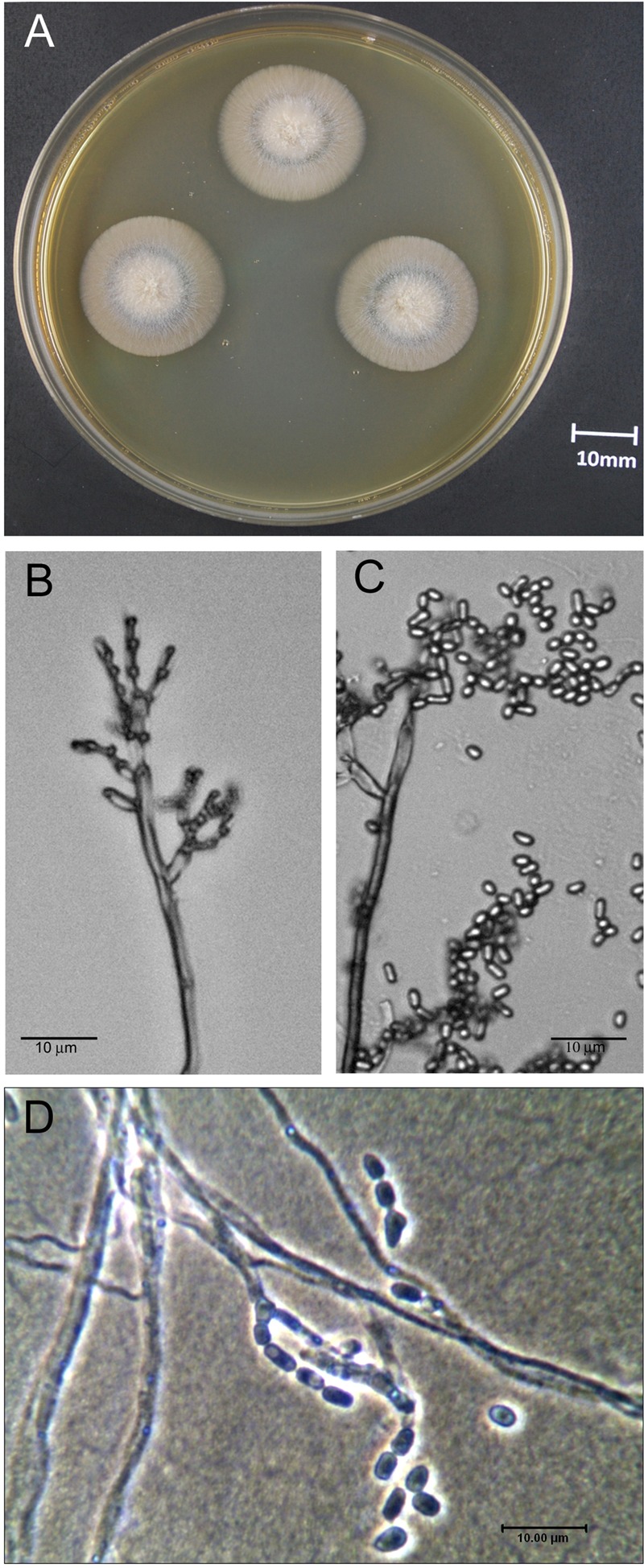
**Morphological characteristics of the mycorrhizal fungus *Oidiodendron maius* var. maius strain Om19 isolated from hair roots of *Rhododendron fortunei*. (A)** Colony of Om19 cultured on malt extract agar (MEA) medium for 14 days. **(B)** Dematiaceous conidiophores of Om19. **(C)** Cylindrical arthroconidia of Om19. **(D)** Cylindrical arthroconidia with one nucleus.

The Om19 formed a thin gray-white colony after incubation in the dark at 25°C for 14 days on MEA medium. Diameter of the colony ranged from 20 to 25 mm (**Figure 1A**). Hyphae of the strain were smooth and transparent, diameter of the hyphae ranged from 1.2 to 2.0 μM. When cultured on 20% potato dextrose broth, tall, and erect conidiophores bearing a head of divergent, branched undulating chains of conidia were viewed under microscope (**Figure 1B**). Conidia had thin-walled, hyaline, subglobose to elongated spores (**Figure 1C**) that appeared to have a single nucleus per spore (**Figure 1D**).

### Molecular Characterization

**FIGURE 2:**
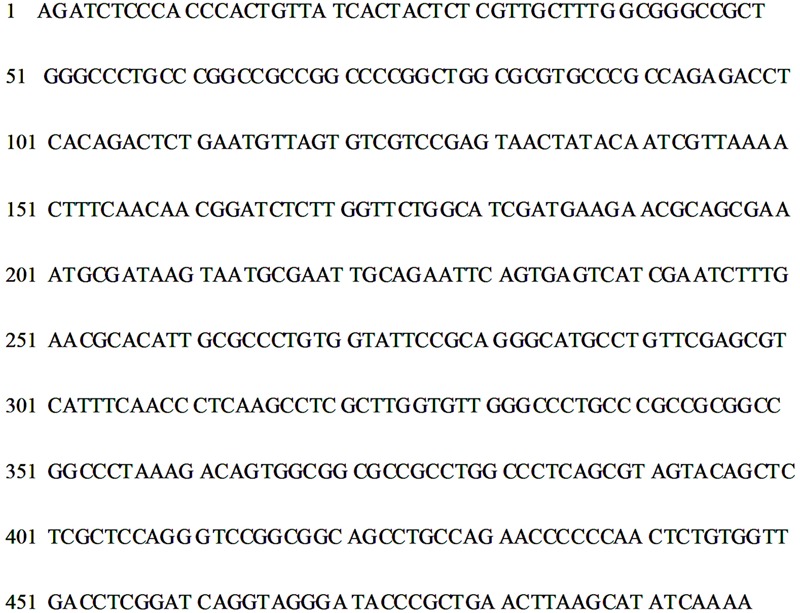
**ITS rDNA sequence of mycorrhizal fungus *O.maius* var. maius strain Om19 isolated from hair roots of *R. fortunei***.

**FIGURE 3:**
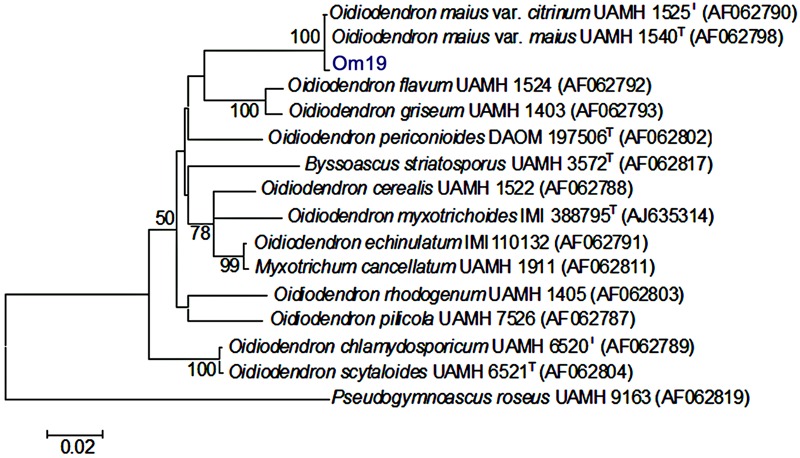
**Neighbor-joining phylogenetic tree based on ITS rDNA sequence data of mycorrhizal fungus *O. maius* var. maius strain Om19 isolated from hair roots of *R. fortunei*, along with known ericoid endophytes and selected fungal species from GenBank with high sequence similarity.** Numerical values above the branches indicate bootstrap percentiles from 1000 replicates. Bootstrap numbers over 50% are indicated. Horizontal branch lengths are proportional to the scale of substitutions.

Molecular analysis of Om19 resulted in a 457 bp ITS rDNA sequence (**Figure 2**). The ITS rDNA sequence was compared to the available sequences obtained by BLAST from the GenBank database. The sequence has 99.8% identity to the sequence of *O. maius* var. *citrinum* UAMH 1525 and *O*. *maius* UAMH 1540. Thus, the strain was placed in the cluster with *O. maius* var. *citrinum* UAMH 1525 and *O*. *maius* UAMH 1540, which is supported by a bootstrap of 100% (**Figure 3**). Both *O. maius* var. *citrinum* UAMH 1525 and *O*. *maius* UAMH 1540 were identified by Barron (1962). The former was isolated from a cedar bog, Canada and the latter was isolated from peat soil in Canada. The Om19 has 91.6 and 92.9% similarity to the other *Oidiodendron* species and also to *Myxotrichum cancellatum* UAMH1991.

### Mycorrhizae Synthesis

**FIGURE 4:**
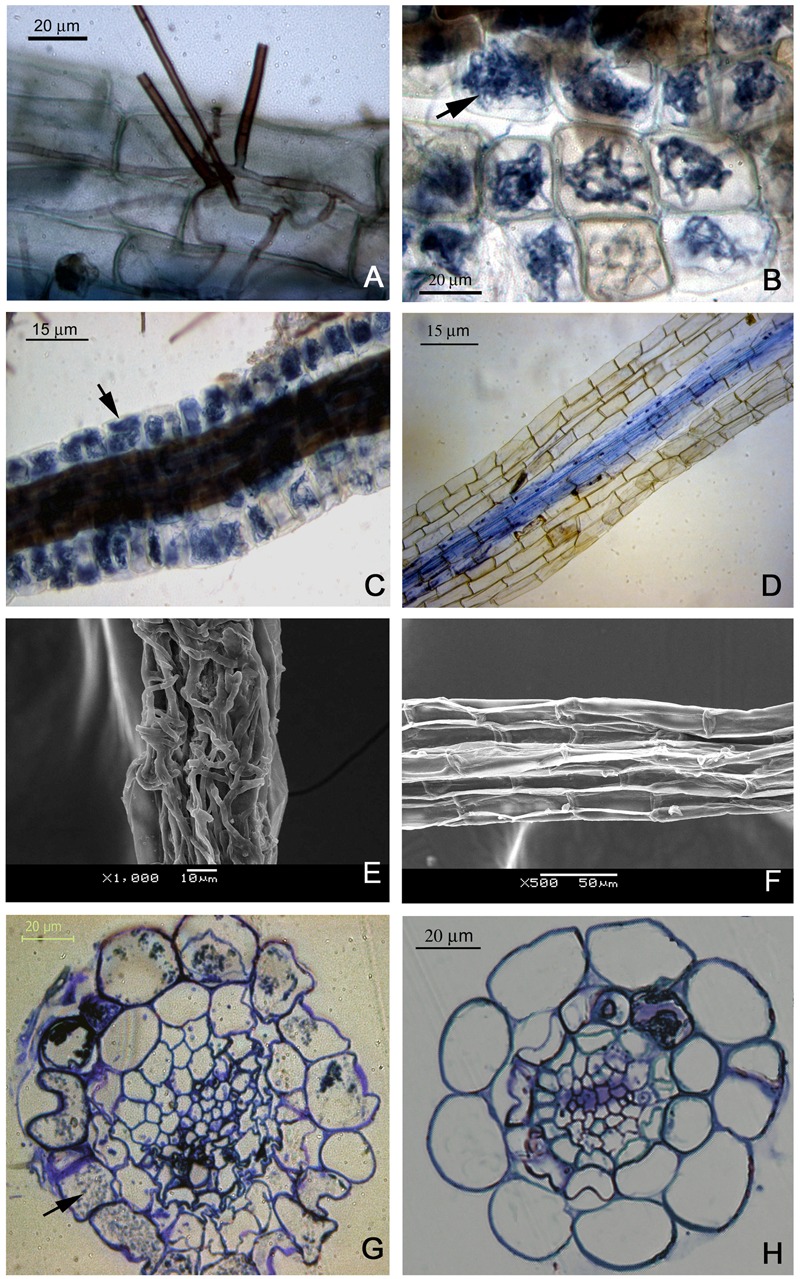
**Microscopic observation of *R. fortunei* roots inoculated or not with mycorrhizal fungus *O. maius* var. maius strain Om19. (A)** A root was infected by Om19 hyphae after inoculation. **(B)** Hyphal growth inside epidermal cells of roots from inoculated plants. **(C)** Hyphae proliferation through all epidermal cells of a root. **(D)** Roots of control seedlings (without inoculation) with no mycorrhizal colonization. **(E)** SEM showing mycelia surrounding roots of inoculated seedlings. **(F)** No mycelia were visible on the surface of control roots. **(G)** Transverse section of a hair root from an inoculated seedling showing hyphal growth in the cortical cells and some distorted cells. **(H)** Transverse section of a hair root from a control seedling showing no hyphal growth.

Microscopic examination of inoculated *R. fortunei* seedlings showed that roots were infected by hyphae (**Figure 4A**); the intracellular hyphal growth was observed in epidermal cells (**Figure 4B**). Some root epidermal and cortical cells completely filled with mycelium after inoculation (**Figure 4C**). The mean percent root length colonized by Om19 ranged from 65 to 72%. There were no fungal structures in root cortical cells of control seedlings (**Figure 4D**). SEM observation also showed that mycelia heavily surrounded roots of seedlings inoculated with Om19 (**Figure 4E**), but the surfaces of control roots were clear (**Figure 4F**). Transverse section of a hair root from a plant inoculated with Om19 showed dense hyphal growth in epidermal cells and a few cortical cells. Some of the epidermal cells in roots from plants inoculated with Om19 appeared to be deformed (**Figure 4G**). Transverse section analysis of the control root showed no hyphal growth inside root cells (**Figure 4H**).

Seedlings inoculated with Om19 were almost two times larger compared to the controls (**Table 1**). Inoculated plants had more roots, greater root length, longer shoots and greater shoot and root fresh weight (**Table 1**).

**Table 1:** Growth of *Rhododendron fortunei* seedlings inoculated or not with isolated ericoid mycorrhizal fungus Om19 for 2 months in a culture room.

Treatment	Root no.	Mean root length (mm)	Root fresh weight (mg)	Leaf no.	Shoot length (mm)	Shoot fresh weight (mg)
Inoculated	7.8^∗∗^	23.2^∗∗^	5.0^∗∗^	11.8	22.3^∗∗^	43.0^∗∗^
Uninoculated	3.5	12.2	2.0	10.0	11.3	20.0

*^∗∗^ indicate significant difference between seedlings inoculated and uninoculated with Om19 at *P* < 0.01 level (*n* = 10).*

### Plant Growth and N Uptake

**FIGURE 5:**
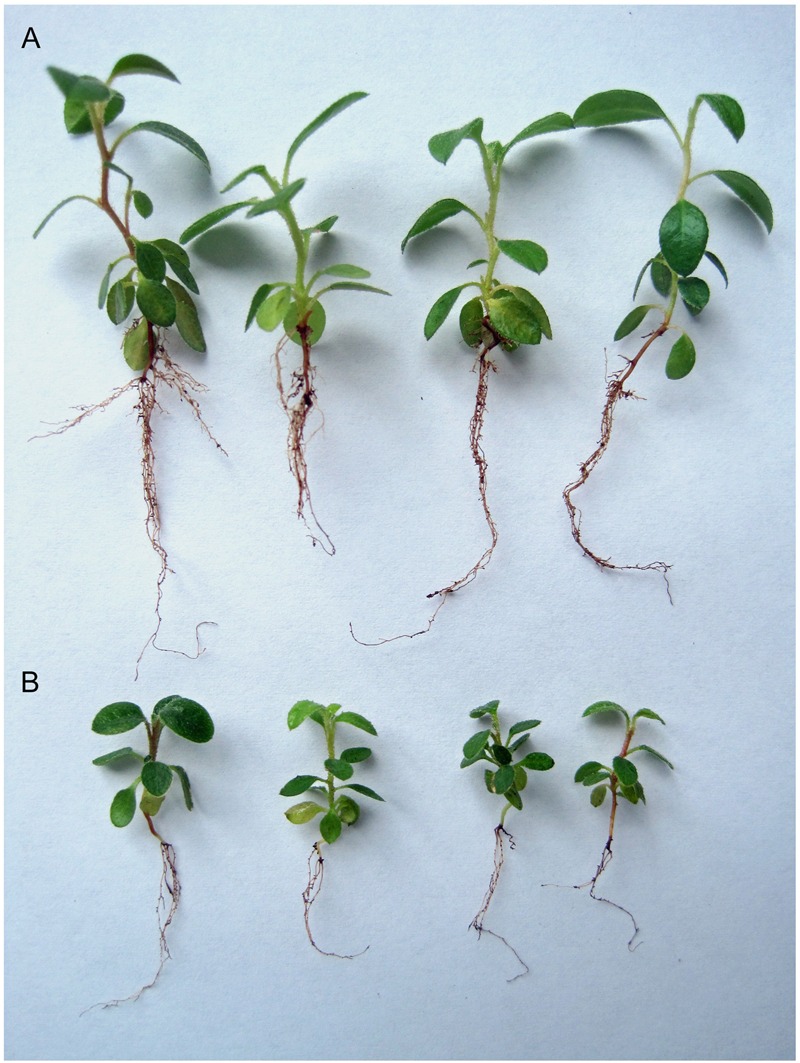
**Seedling of *R. fortunei* inoculated **(A)** or not **(B)** with mycorrhizal fungus *O. maius* var. maius strain Om19 and grown in a peat-based substrate for 2 months**.

When *R. fortunei* seedlings were grown in a peat-based substrate for 2 months, substrate pH ranged from 4.9 to 5.2 across all treatments. Similar to the mycorrhizae synthesis experiment, roots inoculated with Om19 were colonized, and the percent root length colonization ranged from 65 to 72%. The Om19-colonized seedlings grew substantially larger than controls (**Figure 5**). After 2 months of growth, seedlings inoculated with Om19 measured 105% greater fresh weight and 84% greater dry weight than the control seedlings (**Table 2**). Inoculation with Om19 had no significant influence on N concentrations in seedlings, however, total N of inoculated plants was 61% greater than the controls.

**Table 2:** Fresh and dry weights, tissue nitrogen (N) concentration, and total N of 30 *R. fortunei* seedlings grown for 2 months in a peat-based substrate inoculated or not with mycorrhizal fungus Om19.

Treatment	Fresh weight (mg)	Dry weight (mg)	N conc. (%)	Total N (mg)
Inoculated	1,290.0^∗∗^	187.3^∗∗^	1.5	2.9^∗^
Uninoculated	630.0	101.9	1.8	1.8

*^∗^ and ^∗∗^ indicate significant difference between seedlings inoculated and uninoculated with Om19 at *P* < 0.05 and *P* < 0.01 levels (*n* = 5).*

**FIGURE 6:**
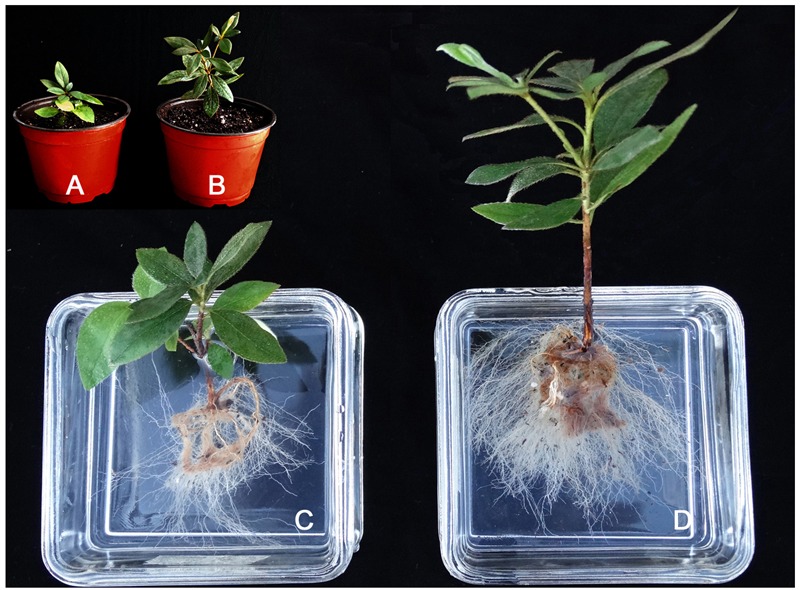
**Growth of Formosa azaleas (*Rhododendron indica* ‘Formosa’) inoculated or not with mycorrhizal fungus *O. maius* var. maius strain Om19, and grown in a shaded greenhouse for 4 months.** Containers with uninoculated **(A)** and inoculated plants **(B)**. Uninoculated **(C)** and inoculated **(D)** plants removed from substrate and roots submerged in water.

Results from the greenhouse trial using commercial production practices showed that Om19 inoculation promoted the growth of rooted cuttings of Formosa azalea (**Figure 6**). Om19-inoculated Formosa azalea plants had more leaves (23) and were taller (16 cm) compared to control plants (14 leaves and 10 cm tall). Furthermore, plants inoculated with Om19 appeared to have more abundant roots compared to the controls (**Figure 6**).

### qRT-PCR Analysis

**FIGURE 7:**
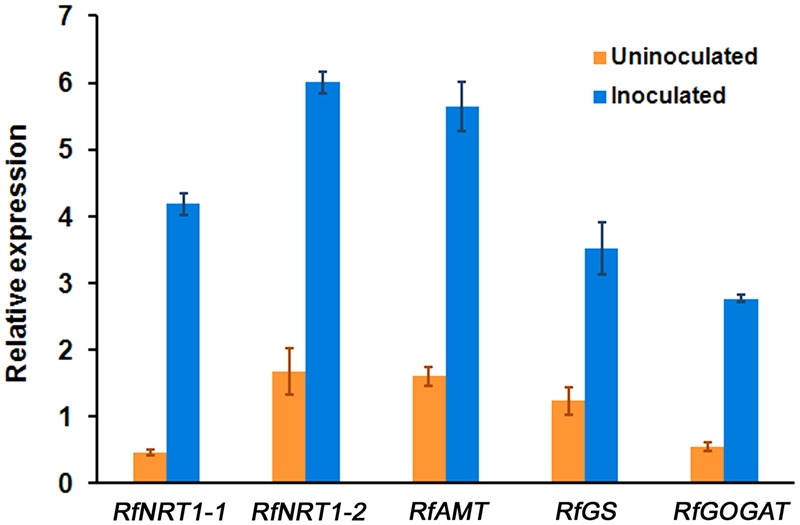
**The expression level of five genes (*RfNRT1-1, RfNRT1-2, RfAMT, RfGS*, and *RfGOGAT*) related to N-uptake and metabolism in roots of *R. fortunei* seedlings 2 months after being inoculated with a mycorrhizal fungus *O. maius* var. maius strain Om19.** The expression levels were normalized based on the expression of the internal control gene and corresponding gene expressed in the control seedlings (seedlings before Om19 inoculation). The bars represent standard errors of three replicates (*n* = 3).

The Om19 colonized the roots of *R. fortunei* seedlings with the percent root length colonization similar to the mycorrhizae synthesis experiment. The qRT-PCR analysis of five genes related to N uptake and metabolism showed that all were increasingly expressed in roots of *R. fortunei* seedlings colonized by Om19 compared to those uninoculated controls (**Figure 7**). The highest expression was *RfNRT1-1*, almost a ninefold increase compared to its counterpart in the control seedlings. The expression of *RfNRT1-2* was 3.5, *RfAMT* was upregulated more than threefold. *RfGS* expression was 2.8-fold higher, and the expressions of *RfGOGAT* was fivefold.

## Discussion

The present study isolated a new *O. maius* strain called Om19 from hair roots of *R. fortunei* grown under mixed pine forests in east China. The Om19 was able to colonize roots of *R. fortunei*, and the colonization improved the growth of both *R. fortunei* and Formosa azalea. Investigation of the expression of genes related to N uptake (*RfAMT, RfNRT1-1*, and *RfNRT1-2*) and N metabolism (*RfGS* and *RfGOGAT*) showed that they were highly upregulated in Om19-colonized roots of *R. fortunei*. Total N of inoculated plants was significantly greater and dry weights were much higher than in the non-colonized control seedlings. The Om19 is promising and could potentially be used as a microbial agent for improving *Rhododendron* and possibly blueberry production.

### Isolate Identification

*Rhododendron fortunei* is native to mountainous areas in east China (Liberty Hyde Bailey Hortorum, 1976). The Huading Forest Park is one of the centers of *R. fortunei* origin. We were able to identify 84 isolates from five plant roots. As indicated by Zhang et al. (2010) and Tian et al. (2011), *R. fortunei* roots harbor a rich microbial ecosystem in their endogenous habitat.

The isolated Om19 reproduced asexually by forming conidia with a single haploid nucleus (**Figure 1**) and was morphologically similar to *O. maius* as described by Barron (1962) and Rice and Currah (2005). ITS rDNA analysis showed a high degree of sequence identity (99.8%) to *O. maius* as reported by Sigler and Gibas (2005) and Zhang et al. (2009) and placed it in the *O. maius* cluster in the neighbor-joining phylogenetic tree (**Figure 3**). It thus was named as *O. maius* Om19.

The Om19 can form mycorrhizae with roots of *R. fortunei.* Root epidermal and cortical cells were filled with mycelium, and transverse section of a hair root showed hyphal growth in epidermal and cortical cells (**Figure 4**). Seedlings colonized by Om19 were significantly larger with more roots (**Table 1**; **Figures 5** and **6**). Our results concurs with those of Jansa and Vosatka (2000), who documented that ERM fungi promoted growth of *Rhododendron* microcutting in peat-based substrates. Yin et al. (2010) also reported that *R. fortunei* plants inoculated with an EMR fungus grew better than the uninoculated controls. These results support our hypothesis that valuable *O. maius* strains could be isolated from hair roots of understorey *R. fortunei* in Chinese forest parks.

### Plant N Uptake and Growth

This study evaluated N uptake and seedling growth of *R. fortunei* inoculated with Om19 under well controlled environmental conditions. Total N absorbed by Om19-colonized seedlings was 61% greater than the control seedlings. Considering the fact that the readily available N in the substrate was NO_3_^-^ and that the pH of the substrate ranged from 4.9 to 5.2, the higher total N value in the Om19-colonized plants may suggest that the mycelium of Om19 could act in a similar function as AM fungi (Gomez et al., 2009; Tian et al., 2010) by absorbing NO_3_^-^, converting it into arginine, and releasing ammonium to plants. Ammonium release would increase *AMT* expression and also trigger plant GS and GOGAT activities. As shown in **Figure 7**, the expression of *RfAMT* increased more than threefold in Om19-colonized roots. Genes in the family of *AMT* varied from 6 to 14, depending plant species; *AMT1-1* and *AMT1-3* were reported to contribute to 30–35% of ammonium transport in plants (Masclaux-Daubresse et al., 2010). Whether or not the *RfAMT* identified in this study belongs to *AMT1-1, AMT1-3*, or other *AMT* requires further investigation. Concomitantly, the expression of *RfGS* increased 2.7 and *RfGOGAT* 5.1-fold in Om19-inoculated roots (**Figure 7**). GS is a key metabolic enzyme that synthesizes glutamine from glutamate, leading to the entrance of organic N in cellular metabolic pathways such as the biosynthesis of amino acids, nucleic acids, and complex polysaccharides. As a result, irrespective of low pH in the substrates, Om19 colonized roots were able to take up more applied N and improve plant growth.

Besides Om19-mediated uptake of NO_3_^-^, *Rhododendron* seedlings *per se* could directly absorb NO_3_^-^ from the substrate. This is because NO_3_^-^ was the readily available N in the peat-based substrate and both *RfNRT1-1* and *RfNRT1-2* were highly upregulated in Om19-colonized seedlings (**Figure 7**). As mentioned previously, plants have LATS and HATS for NO_3_^-^ uptake. In general, the LATS consists of the *NRT1* family and the HATS comprises the *NRT2* family (Sun and Zheng, 2015). There are 53 *NRT1* genes and 7 *NRT2* genes in *Arabidopsis* (Tsay et al., 2007). Recent studies showed that *NRT1.1* is actually a dual-affinity transporter regulating NO_3_^-^ uptake by changing its affinity for NO_3_^-^ depending on the availability of NO_3_^-^ in the soil (Tsay, 2014; Sun and Zheng, 2015). Since the readily available NO_3_^-^ was limited in the peat-based substrate, *NRT1.1* might play an important role in NO_3_^-^ absorption by changing its affinity for NO_3_^-^. At the present, we are not completely certain if either *RfNRT1-1* or *RfNRT1-2* in *R. fortunei* plays the same roles as *NRT1.1* in *Arabidopsis*. The expression of *RfNRT1-1* and *RfNRT1-2* does suggest that NRTs were active in Om19-colonized roots.

The next question is why the upregulation of *RfNRT1-1* and *RfNRT1-2* was greater in Om19-colonized roots than in control roots. One explanation could be that Om19-mediated N uptake enhanced GS/GOGAT cycle (Lea and Forde, 1994), increased Rubisco activity (Masclaux-Daubresse et al., 2010), and elevated NRT gene expression, thus NO_3_^-^ uptake. Wirth et al. (2007) showed that upregulation of NRTs (*AtNRT2.1* and *AtNRT 1-1*) was related to the concentration of glucose 6-phosphate. The direct coupling of NO_3_^-^ assimilation and photosynthesis in chloroplasts is energy efficient and is known as nitrate photoassimilation (Searles and Bloom, 2003). Additionally, several NRT genes were reported to play dual nutrient transport/signaling roles, called transceptors, sensing N availability in soil and regulating N uptake and allocation in plants (Gojon et al., 2011; Krapp et al., 2014; Zhang et al., 2014). In a study of NRT gene expression in tomato plants, the expression of *NRT2;3* was higher in AM-colonized tomato roots than in controls; this was explained as AM-colonization positively affecting nitrate uptake from soil and nitrate allocation to the plant partner (Hildebrandt et al., 2002). The increased uptake of NO_3_^-^ may also improve plant absorption of other ions. For example, a sevenfold increase in N uptake by rhododendron (*Rhododendron* ‘H-1 P.J.M.’) was associated with a threefold–fourfold increase in the uptake rate of phosphorus, potassium, and sulfur, and ∼twofold increase in the uptake rate of magnesium and calcium (Scagel et al., 2008). Similar results were observed in blueberry cultivars inoculated with ERM fungi (Scagel, 2005).

The higher level of total N absorbed by Om19-colonized plants (**Table 2**) may also suggest that Om19 might enzymatically degrade organic N from substrate. A recent study showed that *O. maius* symbionent expressed a full complement of plant cell wall-degrading enzymes in symbiosis, suggesting its saprotrophic ability in sphagnum peat (Kohler et al., 2015). ERM fungi are able to gain access to polymeric sources of N in the form of peptides, pure proteins, or protein-polyphenol complexes (Smith and Read, 2008). The Klasmann peat contains 0.89% N, the decomposition may release organic N for plant absorption. At this point, whether Om19 could enzymatically degrade organic N from the peat is unknown. Further studies for determining its saprotrophic ability and its practical application to be a microbial fertilizer will be fully explored.

Nevertheless, this study suggests that the Om19 can effectively colonize *R. fortunei*, and the colonization improves plant absorption of NO_3_^-^ under acidic growing conditions. Our results thus disagree with the notion that NO_3_^-^ availability is always negligible in acid soils (Paul and Clark, 1989) but support the claims of Scagel (2005) and Kosola et al. (2007) that NO_3_^-^ is an important N source for plants in the family Ericaceae. ERM colonization under acidic soil conditions probably plays a critical role for plants in the heather family to capture NO_3_^-^-N.

### Practical Application

Plants in the family Ericaceae have some unique characteristics: (1) cortical cells never form root hairs, instead their roots are known as hair roots, (2) they have adapted to soils with low pH and low nutrient status, and (3) roots are commonly associated mycorrhizal fungi, mainly ERM fungi. Increasing evidence suggests that the ability to form symbiotic relationships with ERM fungi is critically important to the success of ericaceous plants in these stressful environments (Read, 1996; Cairney and Meharg, 2003; Perotto et al., 2012). Some of these plants, such as cranberry, bilberry, blueberry, and rhododendrons are economically important horticultural crops. However, production of these crops in a sustainable manner has been challenged.

Commercially these crops are produced in acidic soils or acidic substrates, and chemical fertilizers must constantly be applied. Applied N often is leached or is less available to plants because of acidic growing conditions. N leaching has been an environmental concern in commercial production of horticultural crops (Goulding, 2006). Different strategies have been proposed to reduce N leaching (Chen et al., 2001), but little attention has been given to the microbial fertilizers for improving N use efficiency and reducing nutrient leaching (Scagel, 2005). Berruti et al. (2016) recently documented that AM fungi as natural biofertilizers can positively affect plant growth in both controlled and open-field conditions. The present study shows that isolate Om19 is able to colonize *R. fortunei* in an acidic substrate and effectively use applied NO_3_^-^, resulting in increased plant growth. It is worth noting that Om19-inoculated Formaosa azaleas grown in a greenhouse fertilized with a Peters Professional 20–20–20 had visually larger root systems than uninoculated plants. These results suggest that Om19 may be able to colonize different *Rhododendron* species when plants are grown with a common commercial fertilizer containing different forms of N. Taken together, our results demonstrate that Om19 is a potentially important ERM fungus and can be used as a biofertilizer for improving production of *Rhododendron* and possibly other ericaceous plants.

## Author Contributions

CZ, DP, and JC conceived and designed the experiments. XW conducted the experiments, analyzed the data, and drafted the manuscript. JC participated in data analysis, wrote and revised the manuscript. The final version was approved by all authors.

## Conflict of Interest Statement

The authors declare that the research was conducted in the absence of any commercial or financial relationships that could be construed as a potential conflict of interest.

## References

[B1] Addy H. D. Piercey M. M. Currah R. S. (2005). Microfungal endophytes in roots. *Can. J. Bot.* 83 1–13. 10.1111/j.1574-6941.2002.tb00977.x

[B2] Barron G. L. (1962). New species and new records of *Oidiodendron*. *Can. J. Bot.* 40 589–607. 10.1139/b62-055

[B3] Bergero R. Perotto S. Girlanda M. Vidano G. Luppi A. M. (2000). Ericoid mycorrhizal fungi are common root associates of a Mediterranean ectomycorrhizal plant (*Quercus ilex*). *Mol. Ecol.* 9 1639–1649. 10.1046/j.1365-294x.2000.01059.x 11050558

[B4] Berruti A. Lumini E. Balestrini R. Bianciotto V. (2016). Arbuscular mycorrhizal fungi as natural biofertilizers: let’s benefit from past successes. *Front. Microbiol.* 6:1559 10.3389/fmicb.2015.01559 PMC4717633 26834714

[B5] Biermann B. Linderman R. G. (1981). Quantifying vesicular-arbuscular mycorrhizae: a proposed method towards standardization. *New Phytol.* 87 63–67. 10.1111/j.1469-8137.1981.tb01690.x

[B6] Bonfante P. Genre A. (2010). Mechanisms underlying beneficial plant–fungus interactions in mycorrhizal symbiosis. *Nat. Commun.* 1 48 10.1038/ncomms1046 20975705

[B7] Bonfante-Fasolo P. Gianinazzi-Pearson V. (1979). Ultrastructural aspects of endomycorrhiza in the Ericaceae. *New Phytol.* 83 739–744.

[B8] Bougoure D. S. Cairney J. W. G. (2005). Fungi associated with hair roots of *Rhododendron lochiae* (Ericaceae) in an Australian tropical cloud forest revealed by culturing and culture-independent molecular methods. *Environ. Microbiol.* 7 1743–1754. 10.1111/j.1462-2920.2005.00919.x 16232289

[B9] Bucking H. Kafle A. (2015). Role of arbuscular mycorrhizal fungi in the nitrogen uptake of plants: current knowledge and research gaps. *Agronomy* 5 587–612. 10.3390/agronomy5040587

[B10] Cairney J. W. G. Meharg A. A. (2003). Ericoid mycorrhiza: a partnership that exploits harsh edaphic conditions. *Eur. J. Soil Sci.* 54 735–740. 10.1046/j.1351-0754.2003.0555.x

[B11] Chen J. Huang Y. Caldwell R. D. (2001). Best management practices for minimizing nitrate leaching from container-grown nurseries. *ScientificWorldJournal* 1 96–102. 10.1100/tsw.2001.99 12805865 PMC6134969

[B12] Daghino S. Martino E. Perotto S. (2016). Model system to unravel the molecular mechanisms of heavy metal tolerance in the ericoid mycorrhizal symbiosis. *Mycorrhiza* 26 263–274. 10.1007/s00572-015-0675-y 26710764

[B13] Domsch K. H. Gams W. Anderson T. H. (1980). *Compendium of Soil Fungi* Vol. 1 London: Academic Press.

[B14] Economou A. Read P. (1984). In vitro shoot proliferation of Minnesota deciduous azaleas. *Hortscience* 19 60–61.

[B15] Gardes M. Bruns T. D. (1993). ITS primers with enhanced specificity for basidiomycetes – application to the identification of mycorrhizae and rusts. *Mol. Ecol.* 2 113–118. 10.1111/j.1365-294X.1993.tb00005.x 8180733

[B16] Gojon A. l. Krouk G. Perrine-Walker F. Laugier E. (2011). Nitrate transporter(s) in plants. *J. Exp. Bot* 62 2299–2308. 21239382 10.1093/jxb/erq419

[B17] Gomez S. K. Javot H. Deewatthanawong P. Torres-Jerez I. Tang Y. Blancaflor E. B. (2009). Medicago truncatula and *Glomus intraradices* gene expression in cortical cells harboring arbuscules in the arbuscular mycorrhizal symbiosis. *BMC Plant Biol.* 9:10 10.1186/1471-2229-9-10 PMC2649119 19161626

[B18] Goulding K. (2006). Nitrate leaching from arable and horticultural land. *Soil Use Manage.* 16 145–151. 10.1111/j.1475-2743.2000.tb00218.x

[B19] Govindarajulu M. Pfeffer P. E. Jin H. R. Abubaker J. Douds D. D. Allen J. W. (2005). Nitrogen transfer in the arbuscular mycorrhizal symbiosis. *Nature* 435 819–823. 10.1038/nature03610 15944705

[B20] Hambleton S. Currah R. S. (1997). Fungal endophytes from the roots of alpine and boreal Ericaceae. *Can. J. Bot.* 75 1570–1581. 10.1139/b97-869

[B21] Hildebrandt U. Schmelze E. Bothe H. (2002). Expression of nitrate transporter genes in tomato colonized by an arbuscular mycorrhizal fungus. *Physiol. Plant* 115 125–136. 10.1034/j.1399-3054.2002.1150115.x 12010476

[B22] Jansa J. Vosatka M. (2000). In vitro and post vitro inoculation of micropropagated *Rhododendrons* with ericoid mycorrhizal fungi. *Appl. Soil Ecol.* 15 125–136. 10.1016/S0929-1393(00)00088-3

[B23] Kohler A. Kuo A. Nagy L. G. Morin E. Barry K. W. Buscot F. (2015). Convergent losses of decay mechanisms and rapid turnover of symbiosis genes in mycorrhizal mutualists. *Nat. Genet.* 47 410–415. 10.1038/ng.3223 25706625

[B24] Kosola K. R. Workmaster B. A. A. Spada P. A. (2007). Inoculation of cranberry (*Vaccinium macrocarpon*) with the ericoid mycorrhizal fungus *Rhizoscyphus ericae* increases nitrate influx. *New Phytol.* 176 184–196. 10.1111/j.1469-8137.2007.02149.x 17803649

[B25] Krapp A. David L. C. Chardin C. Girin T. Marmagne A. Leprince A. (2014). Nitrate transport and signaling in *Arabidopsis*. *J. Exp. Bot.* 65 789–798. 10.1093/jxb/eru001 24532451

[B26] Lea P. J. Forde B. G. (1994). The use of mutants and transgenic plants to studt amnion acid metabolism. *Plant Cell Environ.* 17 541–556. 10.1111/j.1365-3040.1994.tb00148.x

[B27] Liberty Hyde Bailey Hortorum (1976). *Hortus Third: A Concise Dictionary of Plants Cultivated in the United States and Canada*. New York, NY: Macmillon.

[B28] Malagoli P. Lainé P. Le Deunff E. Rossato L. Ney B. Ourry A. (2004). Modeling nitrogen uptake in oilseed rape cv Capitol during a growth cycle using influx kinetics of root nitrate transport systems and field experimental data. *Plant Physiol.* 134 388–400. 10.1104/pp.103.029538 14671012 PMC316318

[B29] Masclaux-Daubresse C. Daniel-Vedele F. Dechorgnat J. Chardon F. Gaufichon L. Suzuki A. (2010). Nitrogen uptake, assimilation and remobilization in plants: challenges for sustainable and productive agriculture. *Ann. Bot.* 105 1141–1157. 10.1093/aob/mcq028 20299346 PMC2887065

[B30] Paul E. A. Clark F. E. (1989). *Soil Microbiology and Biochemistry.* San Diego, CA: Academic Press.

[B31] Perotto S. Martino E. Abba S. Vallino M. (2012). “Genetic diversity and functional aspects of ericoid mycorrhizal fungi,” in *Fungal Associations: The Mycota IX* 2nd Edn. ed. Hock B. (Berlin: Springer-Verlag).

[B32] Phillips J. Hayman D. (1970). Improved procedures for clearing roots and staining parasitic and vesicular-arbuscular mycorrhizal fungi for rapid assessment of infection. *Trans. Br. Mycol. Soc.* 55 158–161. 10.1016/S0007-1536(70)80110-3

[B33] Read D. J. (1996). The structure and function of the ericoid mycorrhizal root. *Ann. Bot.* 77 365–374. 10.1006/anbo.1996.0044

[B34] Rice A. V. Currah R. S. (2005). *Oidiodendron*: a survey of the named species and related anamorphs of *Myxotrichum*. *Stud. Mycol.* 53 83–120. 10.3114/sim.53.1.83

[B35] Rice A. V. Currah R. S. (2006). “*Oidiodendron maius*: saprobe in sphagnum peat, mutualist in ericaceous roots?,” in *Microbial Roots Endophytes* eds Shulz B. J. E. Boyle C. J. C. Sieber T. N. (Berlin: Springer-Verlag) 227–246.

[B36] Rosen C. J. Allan D. L. Luby J. J. (1990). Nitrogen form and solution pH influence growth and nutrition of two *Vaccinium* clones. *J. Am. Soc. Hort Sci.* 115 83–89.

[B37] Scagel C. Bi G. Fuchigami L. H. Regan R. P. (2008). Nitrogen availability alters mineral nutrient uptake and demand in container-grown deciduous and evergreen rhododendron. *J. Environ. Hortic.* 26 177–187.

[B38] Scagel C. F. (2005). Inoculation with ericoid mycorrhizal fungi alters fertilizer use of highbush blueberry cultivars. *HortScience* 40 786–794.

[B39] Schmittgen T. D. Livak K. J. (2008). Analyzing real-time PCR by comparative CT method. *Nat. Protoc.* 3 1101–1108. 10.1038/nprot.2008.73 18546601

[B40] Scoggins H. L. Bailey D. A. Nelson P. V. (2002). Efficacy of the press extraction method for bedding plant plug nutrient monitoring. *HortScience* 37 108–112.

[B41] Searles P. S. Bloom A. J. (2003). Nitrate photo-assimilation in tomato leaves under short-term exposure to elevated carbon dioxide and low oxygen. *Plant Cell Environ.* 26 1247–1255. 10.1046/j.1365-3040.2003.01047.x

[B42] Sigler L. Gibas C. F. C. (2005). Utility of a cultural method for identification of the ericoid mycobiont *Oidiodendron maius* confirmed by ITS sequence analysis. *Stud. Mycol.* 53 63–74. 10.3114/sim.53.1.63

[B43] Smith J. D. (1993). *Uptake and Utilization of Nitrogen Sources by Cranberry Plants (Vaccinium macrocarpon, Ait.).* Madison, WI: University of Wisconsin.

[B44] Smith S. E. Read D. J. (2008). *Mycorrhizal Symbiosis* 3rd Edn. New York, NY: Elsevier.

[B45] Stoyke G. Currah R. S. (1991). Endophytic fungi from the mycorrhizae of alpine ericoid plants. *Can. J. Bot.* 69 347–352. 10.1139/b91-047

[B46] Sun J. Zheng N. (2015). Molecular mechanism underlying the plant NRT1.1 dual-affinity nitrate transporter. *Front. Physiol.* 6:386 10.3389/fphys.2015.00386 PMC4683204 26733879

[B47] Thom C. Church M. B. (1926). *The Aspergilli.* Baltimore, MD: Williams and Wilkons.

[B48] Tian C. Kasiborski B. Koul R. Lammers P. J. Bucking H. Shachar-Hill Y. (2010). Regulation of the nitrogen transfer pathway in the arbuscular mycorrhizal symbiosis: gene characterization, and the coordination of expression with nitrogen flux. *Plant Physiol.* 153 1175–1187. 10.1104/pp.110.156430 20448102 PMC2899933

[B49] Tian W. Zhang C. Q. Qiao P. Milne R. (2011). Diversity of culturable ericoid mycorrhizal fungi of *Rhododendron decorum* in Yunnan, China. *Mycologia* 103 703–709. 10.3852/10-296 21289105

[B50] Tokumasu S. (1973). Notes on Japanese *Oidiodendron* (Japanese microscopic fungi II). *Trans. Mycol. Soc. Jpn.* 14 246–255.

[B51] Tsay Y. F. (2014). Plant science: how to switch affinity. *Nature* 507 44–45. 10.1038/nature13063 24572361

[B52] Tsay Y. F. Chiu C. C. Tsai C. B. Ho C. H. Hsu P. K. (2007). Nitrate transporters and peptide transporters. *FEBS Lett.* 581 2290–2300. 10.1016/j.febslet.2007.04.047 17481610

[B53] Usuki F. Abe J. P. Kakishima M. (2003). Diversity of ericoid mycorrhizal fungi isolated from hair roots of *Rhododendron obtusum* var. *Kaempferi* in a Japanese red pine forest. *Mycoscience* 44 97–102. 10.1007/S10267-002-0086-8

[B54] von Wittgenstein N. J. Le C. H. Hawkins B. J. Ehlting J. (2014). Evolutionary classification of ammonium, nitrate, and peptide transporters in land plants. *BMC Evol. Biol.* 14:11 10.1186/1471-2148-14-11 PMC3922906 24438197

[B55] Wirth J. Chopin F. Santoni V. Vienois G. Tillard P. Krapp A. (2007). Regulation of root nitrate uptake at NRT2-1 protein level in *Arabidopsis thaliana*. *J. Biol. Chem.* 282 23541–23552. 10.1074/jbc.M700901200 17573350

[B56] Xiao G. Berch S. M. (1992). Ericoid mycorrhizal fungi of *Gaultheria shallon*. *Mycologia* 84 470–471. 10.2307/3760201

[B57] Yin L. Zhang C. Yang B. (2010). Characteristics of nitrogen absorbed by ericoid mycorrhizal fungi and impact on growth of *Rhododendron fortunei*. *Sci. Agric. Sin.* 43 868–872.

[B58] Zhang C. Chen Z. Yu F. Yin L. (2010). Effects of colonization with different ericoid mycorrhizal fungi on Rododendron fortunei and selection of superior ERM fungi. *Acta Agric. Shanghai* 26 38–41.

[B59] Zhang C. Yin L. Dai S. (2009). Diversity of root-associated fungal endophytes in *Rhododendron fortunei* in subtropical forests of China. *Mycorrhiza* 19 417–423. 10.1007/s00572-009-0246-1 19396474

[B60] Zhang G. Yi H. Gong J. (2014). The *Arabidopsis* ethylene/jasmonic acid-NRT signal module coordinates nitrate reallocation and the trade-off between growth and environmental adaptation. *Plant Cell* 26 3984–3998. 10.1105/tpc.114.129296 25326291 PMC4247569

