# Supplementary material for: A New Oidiodendron maius Strain Isolated from Rhododendron fortunei and its Effects on Nitrogen Uptake and Plant Growth
Source: Front Microbiol. 2016 Aug 23;7:1327. doi: 10.3389/fmicb.2016.01327 (PMC4993752; doi:10.3389/fmicb.2016.01327)
Supplement: Supplementary file 1 [file Table_1.DOC]

Table S1. Sequence homology of the selected five genes from *Rhododendron fortunei* to those in other plant species based on Nucleotide Collection (nr/nt) database.

| Gene ID | Size  (bp) | Homologous protein | Organism origin | Accession Number | Query cover | E-value | Identity |
| --- | --- | --- | --- | --- | --- | --- | --- |
| *RfAMT* | 1781 | Ammonium transporter 3 member 1-like | *Vitis vinifera* | XM_002272699.2 | 75% | 0.0 | 79% |
|  |  | Ammonium transporter 3 member 1-like | *Vitis vinifera* | XM_002271078.2 | 75% | 0.0 | 78% |
|  |  | Ammonium transporter 3.1-like protein | *Camellia sinensis* | KP338998.1 | 75% | 0.0 | 78% |
|  |  | Ammonium transporter 3 member 1-like | *Eucalyptus grandis* | XM_010027394.1 | 75% | 0.0 | 78% |
|  |  | Ammonium transporter 3 member 3-like | *Sesamum indicum* | XM_011098789.1 | 76% | 0.0 | 77% |
| *RfNRT1-1* | 1878 | NRT1/ PTR FAMILY 2.13 | *Vitis vinifera* | XM_002269304.2 | 85% | 0.0 | 71% |
|  |  | Hypothetical protein | *Citrus clementina* | XM_006438498.1 | 84% | 0.0 | 70% |
|  |  | Proton-dependent oligopeptide transport family protein | *Populus trichocarpa* | XM_006378103.1 | 85% | 0.0 | 69% |
|  |  | Protein NRT1/ PTR FAMILY 2.13-like | *Citrus sinensis* | XM_006483193.2 | 84% | 0.0 | 69% |
|  |  | Protein NRT1/ PTR FAMILY 2.13-like | *Citrus sinensis* | XM_006483192.2 | 84% | 0.0 | 69% |
| *RfNRT1-2* | 1772 | Protein NRT1/ PTR FAMILY 2.13 | *Vitis vinifera* | XM_002269304.2 | 90% | 0.0 | 71% |
|  |  | Hypothetical protein mRNA, complete cds | *Citrus clementina* | XM_006438498.1 | 89% | 0.0 | 70% |
|  |  | Protein NRT1/ PTR FAMILY 2.13-like | *Citrus sinensis* | XM_006483193.2 | 89% | 0.0 | 70% |
|  |  | Protein NRT1/ PTR FAMILY 2.13-like | *Citrus sinensis* | XM_006483192.2 | 89% | 0.0 | 70% |
|  |  | Proton-dependent oligopeptide transport family protein | *Populus trichocarpa* | XM_006378103.1 | 90% | 0.0 | 69% |
| *RfGS* | 876 | Glutamine synthetase leaf isozyme | *Ricinus communis* | XM_002516755.2 | 96% | 0.0 | 83% |
|  |  | Glutamine synthetase leaf isozyme | *Ricinus communis* | XM_015717842.1 | 96% | 0.0 | 83% |
|  |  | Glutamine synthetase leaf isozyme | *Ricinus communis* | XM_015717841.1 | 96% | 0.0 | 83% |
|  |  | Glutamine synthetase precursor (plGS) mRNA | *Juglans nigra* | AF169795.1 | 97% | 0.0 | 83% |
|  |  | Glutamine synthetase leaf isozyme | *Jatropha curcas* | XM_012214101.1 | 96% | 0.0 | 83% |
| *RfGOGAT* | 3855 | Glutamate synthase [NADH] | *Vitis vinifera* | XM_010664684.1 | 99% | 0.0 | 83% |
|  |  | Glutamate synthase [NADH] | *Vitis vinifera* | XM_010664683.1 | 99% | 0.0 | 83% |
|  |  | Glutamate synthase [NADH] | *Vitis vinifera* | XM_002267829.3 | 99% | 0.0 | 83% |
|  |  | Glutamate synthase [NADH] | *Vitis vinifera* | XM_010664682.1 | 99% | 0.0 | 83% |
|  |  | Glutamate synthase [NADH] | *Jatropha curcas* | XM_012234693.1 | 99% | 0.0 | 81% |
