# Supplementary material for: A New Oidiodendron maius Strain Isolated from Rhododendron fortunei and its Effects on Nitrogen Uptake and Plant Growth
Source: Front Microbiol. 2016 Aug 23;7:1327. doi: 10.3389/fmicb.2016.01327 (PMC4993752; doi:10.3389/fmicb.2016.01327)
Supplement: Supplementary file 2 [file Table_2.DOC]

**Table S2 qRT-PCR primers**

| **Reference gene** | **Sequence** |
| --- | --- |
| *EF1α*-F | TGTCATCGATGCTCCTGGAC |
| *EF1α*-R | TCTCGGGTCTGACCACCCTT |
| **Target genes** | **Sequence** |
| *RfNRT1-1*-F | AGTGTTGCCAATGCCCTATTCTTC |
| *RfNRT1-1*-R | ATATCGTTTGTCAGCCAGTTCGG |
|  |  |
| *RfNRT1-2*-F | AAGTTCAACATGGAGCAGGCATC |
| *RfNRT1-2*-R | AGCGAAGGAGGCAACAACAATG |
|  |  |
| *RfAMT*-F | TCCTCCTCATTTCGTATATGTGGTAG |
| *RfAMT*-R | GCGTTGTCTCCTTTGTTCAACC |
| *RfGS*-F | CTTTTGATGGCGTCCTTGAG |
| *RfGS*-R | TCCCACGGTTCCATAAGTGC |
|  |  |
| *RfGOGAT*-F | TGACTCCACACCCTACTGTTCTAC |
| *RfGOGAT*-R | ATGCGATTTCAAACTGACCTTTCC |
